# Low-intensity pulsed ultrasound promotes periodontal regeneration in a beagle model of furcation involvement

**DOI:** 10.3389/fbioe.2022.961898

**Published:** 2022-08-26

**Authors:** Yue Wang, Qingyue Xiao, Wenjie Zhong, Chuangwei Zhang, Yuanyuan Yin, Xiang Gao, Jinlin Song

**Affiliations:** ^1^ College of Stomatology, Chongqing Medical University, Chongqing, China; ^2^ Chongqing Key Laboratory of Oral Diseases and Biomedical Sciences, Chongqing, China; ^3^ Chongqing Municipal Key Laboratory of Oral Biomedical Engineering of Higher Education, Chongqing, China

**Keywords:** furcation involvement, low-intensity pulsed ultrasound, inflammation, regeneration, beagle dog

## Abstract

**Objective:** To evaluate the regeneration potential of periodontitis tissue treated by low-intensity pulsed ultrasound (LIPUS) combined with the guided tissue regeneration (GTR) technique in a beagle model of furcation involvement (FI).

**Background:** Achieving predictable regeneration remains a clinical challenge for periodontitis tissue due to the compromised regenerative potential caused by chronic inflammation stimulation. LIPUS, an FDA-approved therapy for long bone fracture and non-unions, has been demonstrated effective in the *in vitro* attenuation of inflammation-induced dysfunction of periodontal ligament stem cells (PDLSCs), the key cells contributing to periodontal regeneration. However, the *in vivo* effect of LIPUS on periodontitis tissue is rarely reported.

**Methods:** A beagle model of FI was established, and the experimental teeth were randomly assigned into three groups: control group, GTR group, and GTR+LIPUS group. Radiographic examinations were performed, and clinical periodontal parameters were recorded to reflect the periodontal condition of different groups. Histological analyses using H&E and Masson’s staining were conducted to evaluate the periodontal tissue regeneration.

**Results:** LIPUS could enhance new periodontal bone formation and bone matrix maturity in FI after GTR treatment. Moreover, clinical assessment and histomorphometric analyses revealed less inflammatory infiltration and superior vascularization within bone grafts in the LIPUS treatment group, indicating the anti-inflammatory and pro-angiogenic effects of LIPUS in FI.

**Conclusion:** Our investigation on a large animal model demonstrated that LIPUS is a promising adjunctive approach for the regeneration of periodontitis tissue, paving a new avenue for LIPUS application in the field of periodontal regenerative medicine.

## 1 Introduction

Periodontitis is a chronic inflammatory disease of the tooth-supporting structure characterized by the progressive loss of periodontal attachment and alveolar bone (Slots, 2017; Papapanou et al., 2018). As a major cause of tooth loss and a potential contributor to systemic inflammation, periodontitis is becoming a global health concern with severe implications for patients and a high financial burden for healthcare systems throughout the world (Kassebaum et al., 2014; Kinane et al., 2017). During the past decades, a series of approaches including non-surgical and surgical therapies were developed for controlling inflammation and arresting the progression of periodontitis, but reconstruction of the periodontal defect caused by periodontitis remains a clinical challenge in modern dentistry (Kinaia et al., 2011; Dentino et al., 2013). Currently, regenerative treatments based on the theory of guided bone or tissue regeneration (GBR/GTR) are regarded as effective methods for periodontal regeneration (Bowers et al., 2003; Aprile et al., 2020). However, due to the compromised regenerative capability of periodontal tissues under chronic inflammation, traditional regenerative treatments require a long-term healing period, which predisposes patients to various high-risk factors such as secondary infection and poor oral hygiene (Jiang et al., 2018; Xu et al., 2019; Ying et al., 2020). As a consequence, the therapeutic outcome of the diseased teeth is often unpredictable, particularly on posterior multi-rooted teeth, for their complex anatomic structure in the furcation regions which leads to an increased inflammatory burden (Reddy et al., 2015; Dommisch et al., 2020; Rasperini et al., 2020). For a more accurate determination of prognosis, developing an adjunctive therapy that could rescue the impaired regenerative capacity and further accelerate the regeneration process of periodontal tissues is ongoingly pursued in the field of periodontal medicine (Reddy et al., 2015).

Low-intensity pulsed ultrasound (LIPUS) therapy, an FDA-approved technique, has been widely used for the treatment of long bone fractures with non-unions or delayed healing in the field of orthopedics (Busse et al., 2009; Jiang et al., 2019). As a non-invasive treatment modality, it could trigger biological responses in bone tissue such as osteoblast differentiation, extracellular matrix remodeling, and angiogenesis via acoustic wave-mediated rhythmic mechanical stimulation (Harrison et al., 2016; Matsumoto et al., 2018; Jiang et al., 2019; Kang et al., 2019; Ding et al., 2020). In recent years, several studies reported that in addition to its positive impact on bone healing, LIPUS therapy also showed great potential for the regeneration of periodontal tissue under inflammatory conditions, thus attracting wide interest in the dental field (Tanaka et al., 2015; Zhang et al., 2020; Xu et al., 2021). For example, it has been demonstrated that daily treatment of LIPUS (90 mW/cm^2^, 1.5 MHz) could rescue the impaired osteogenic capacity of human periodontal ligament stem cells (hPDLSCs) caused by lipopolysaccharide (LPS) through the activation of autophagy and suppression of endoplasmic reticulum stress (Li et al., 2020; Ying et al., 2020; Xia et al., 2021). Moreover, the expression of LPS-induced inflammatory factors like TNF-α, IL-1β, and IL-6 in macrophages and hPDLSCs could be inhibited by LIPUS via downregulation of the nuclear factor-κB (NF-κB) signaling pathway, indicating the anti-inflammatory effect of LIPUS (Xu et al., 2021). Despite some encouraging results, the majority of studies could only provide *in vitro* evidence. Even some researchers explored the effect of LIPUS therapy on rodent models with periodontitis, but the data based on small animal experiments are still not sound enough to support the therapeutic effect of LIPUS on periodontal regeneration under inflammatory conditions due to the great differences in physiological, pathological, immunological, and anatomical structures of periodontal tissues between rodents and human beings (Prabhakar, 2012; Ribitsch et al., 2020). Therefore, preclinical studies on large animals are in great demand to promote the clinical translation of LIPUS from bench to bedside.

A beagle model of furcation involvement (FI), one of the most common sequelae of periodontitis, was established in our study. To investigate the effect of LIPUS on periodontal regeneration, the experimental teeth were randomly assigned into three groups: the control group, the GTR group, and the GTR+LIPUS group. Our findings based on clinical assessment, radiographic examination, and histopathological analyses suggest that LIPUS could accelerate new periodontal bone formation in the FI model treated by GTR, with the role of anti-inflammation and pro-angiogenesis in the root furcation region, which is believed to open a new avenue for LIPUS application in the field of periodontal regenerative medicine.

## 2 Materials and methods

### 2.1 Animals and design

The experimental protocol was approved by the Ethics Committee of Stomatological Hospital of Chongqing Medical University (CQHS-REC-2021 LSNo.50), and all animals were provided by the Experimental Animal Center of Chongqing Medical University. All surgical procedures were performed under general anesthesia. Five beagles, aged from 12 to 18 months and weighed from 7 to 11 kg, were kept in separate cages under a 12-h light/dark cycle and were fed with standard food and water.

For the experimental teeth, three premolars were randomly selected from the bilateral mandibular of each beagle. Then, the class II furcation defects were created at experimental tooth sites. To avoid the effects of LIPUS treatment on the adjacent teeth, the single premolar of the unilateral mandibular was included in the LIPUS+GTR group. Therefore, 15 mandibular premolars were assigned to three groups: the control group, the GTR group, and the GTR+LIPUS group. The experimental design and timeline are shown in Supplementary Figure S1.

### 2.2 Surgical procedures

#### 2.2.1 Establishment of an experimental class II FI model

The establishment of an experimental class II FI model was performed with reference to the previously described methods (Wikesjo et al., 1994; Wikesjo et al., 2003; Struillou et al., 2010). Briefly, periodontal scaling was conducted on all experimental teeth 2 weeks prior to the surgery. Then, the beagles were generally anesthetized by intramuscular injection of Su-Mian-Xin II (0.1 ml/kg) and diazepam (0.3 ml). The surgical region was rinsed with 2% H_2_O_2_ after general anesthesia, and a mucoperiosteal flap was elevated on the buccal side to expose the furcation area of premolars. A “U”-shaped defect with 3 mm in the bucco-lingual direction, 5 mm in the mesial–distal direction, and 6 mm in the crown–root direction was created at the furcation region using a dental drill. A reference notch was made on the root surface at the bottom of the defect for histomorphometric measurement. The 0.25-mm stainless-steel ligature was tied at the cervical margin of premolars, and the knot was placed at the furcation region. Finally, the surgical area was flushed with saline and closed with a 4–0 silk ligament. After surgery, all beagles were intramuscularly injected with penicillin for 3 days, feeding on sugar and mashed potatoes for 8 weeks. To confirm the successful establishment of experimental class II furcation involvement, bone loss at the furcation was examined by X-ray after modeling. Moreover, clinical periodontal parameters, including bleeding index (BI), probing depth (PD), and attachment loss (AL), were recorded to assess the periodontal status.

#### 2.2.2 GTR and LIPUS treatments

To exclude the occlusal impact on periodontal treatment, crown resection was performed on the experimental teeth in all groups, followed by root canal therapy and removal of stainless-steel ligatures. At the end of the eighth week, the experimental teeth in all groups received root scaling. One week later, the GTR surgery was conducted on both the GTR group and GTR+LIPUS group by an experienced surgeon under standard procedures. In the condition of physiological saline cooling, the autologous bone graft (around 5 × 5 × 4 mm^3^) was harvested from the mandibular first molar area via a fissure bur and osteotome. Then, it was smashed by scissors and implanted into the furcation defect. The polytetrafluoroethylene (PTFE) membrane was reshaped to cover the defect area with a proper margin. At last, the flap was properly repositioned and sewn up with 4–0 silk sutures. To prevent post-operative infection, intramuscular injections of penicillin (800,000 IU/day for 1 week) were prescribed, and 1.5% hibitane solution was used daily on the operated area. Moreover, the diet was adjusted to soft food during the healing time, and sutures were removed on the seventh day after surgery. The GTR+LIPUS group received LIPUS treatment after suture removal under general anesthesia. LIPUS irradiation was performed via an ultrasonic probe connected to the ultrasonic therapeutic device (National Engineering Research Center of Ultrasound Medicine, Chongqing) for 20 min per day over a period of 6 weeks. The parameters of LIPUS were set as follows: frequency at 1.5 MHz, wave width at 200 μs, pulse ratio at 1.0 kHz, and intensity at 90 mW/ cm^2^ (Wang et al., 2018). Meanwhile, the GTR group received the same treatment as the GTR+LIPUS group without switching on the ultrasound generator, and the control group only received routine cleaning.

### 2.3 Clinical assessment

Clinical periodontal parameters, including bleeding index (BI), probing depth (PD), and attachment level (AL), were recorded to reflect the periodontal condition of different groups. BI scores were recorded on a scale from 0 to 5 (Mazza et al., 1981). PD was measured using a periodontal probe. AL was measured by the distance from the cemento-enamel junction to the bottom of the periodontal sulcus. A separate clinical assessment was performed at the end of the FI model establishment and LIPUS treatment to evaluate the eventual therapeutic effects among different groups. Moreover, the examiner was blinded to the study groups.

### 2.4 Histological and histomorphometric analyses

After a six-week LIPUS treatment for experimental teeth in the GTR+LIPUS group, all beagles were sacrificed to collect the implanted PTFE membranes and samples in the control, GTR, and GTR+LIPUS groups. The collected PTFE membranes were fixed in 2.5% glutaraldehyde solution for further observation under a scanning electron microscope (SEM, Model JSM-7500F). The harvested samples from the experimental area were fixed in 4% neutral formaldehyde solution. Afterward, the fixed samples were decalcified with 10% EDTA solution and embedded in paraffin. The 5-μm-thick paraffin sections were prepared for H&E and Masson’s trichrome staining to evaluate the periodontal tissue regeneration. The proportion of new bone area (NBA/DA), cementum (NC/NV), and ligament formation (NP/NV) was analyzed using Image J software. Referring to ISO 10993–6 (2016), local lymphocyte infiltration and neovascularization were semi-quantitatively analyzed (Supplementary Table S1, Supplementary Table S2).

### 2.5 Statistical analysis

Statistical analysis of experimental data was performed via SPSS 13.0. The normality of the data distribution and the homogeneity of variance were evaluated by the Shapiro–Wilk test and Levene test, respectively. Comparisons among different groups were conducted with one-way ANOVA, and multiple comparisons were carried out with the LSD test. All data were presented as the mean ± SD. The differences were considered significant at *p < 0.05.

## 3 Results

### 3.1 FI model establishment

**FIGURE 1:**
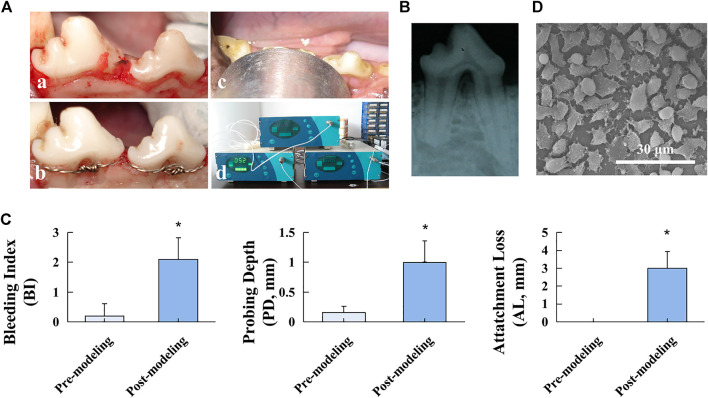
Beagle FI model establishment. **(A)** Modeling procedures: a. flap elevation, b. establishment of the “U”-shaped defect and stainless-steel ligature fastening, c. LIPUS treatment, and d. ultrasonic therapeutic device. **(B)** Postoperative radiographic observation. **(C)** Clinical assessment of BI, PD, and AL before and after the modeling procedure. Data are presented as mean ± SD. *p < 0.05. **(D)** SEM image of the PTFE membrane.

To resemble the pathophysiology of FI and obtain reliable evaluation outcomes, a typical and stable FI model was established on beagles (Figure 1A). All beagles survived throughout the experimental procedures. As shown in Figures 1A,B, the radiolucent region at the root furcation area was observed post modeling, suggesting the successful FI model establishment. Furthermore, stimulation of ligature and sweet/sticky food led to poor periodontal hygiene, as indicated by the significantly increased clinical indicators, including BI, PD, and AL, after modeling (*p* < 0.05, Figure 1C), which facilitated the formation of inflammation conditions in periodontal tissue. The surface of the barrier membrane was observed under SEM (Figure 1D). The SEM images revealed the highly spread adherent cells with filopodia extending to adjacent cells, suggesting favorable biocompatibility of the PTFE membrane.

### 3.2 Clinical assessment

**FIGURE 2:**
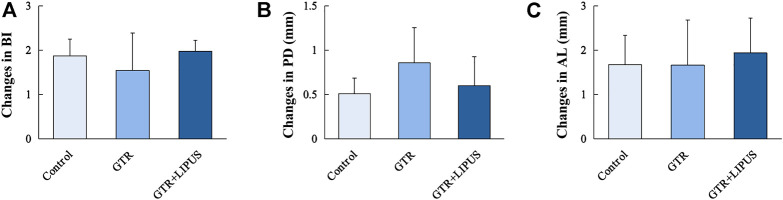
Clinical periodontal parameters of different groups. BI **(A)**, PD **(B)**, and AL **(C)** of the control group, GTR group, and GTR+LIPUS group. Data are presented as mean ± SD.

Clinical assessment revealed the general periodontal status after GTR with or without LIPUS treatment. As shown in Figure 2, the severity of periodontal bleeding, probing depth, and attachment loss was relieved in all groups, but the changes in PD, AL, and BI values were not significant among the three groups (*p* > 0.05). The improved results of the clinical assessment indicated the efficacy of routine root scaling prior to GTR and LIPUS treatments. A more accurate histological evaluation could be achieved under identical periodontal hygiene conditions.

### 3.3 Histological and histomorphometric analyses

**FIGURE 3:**
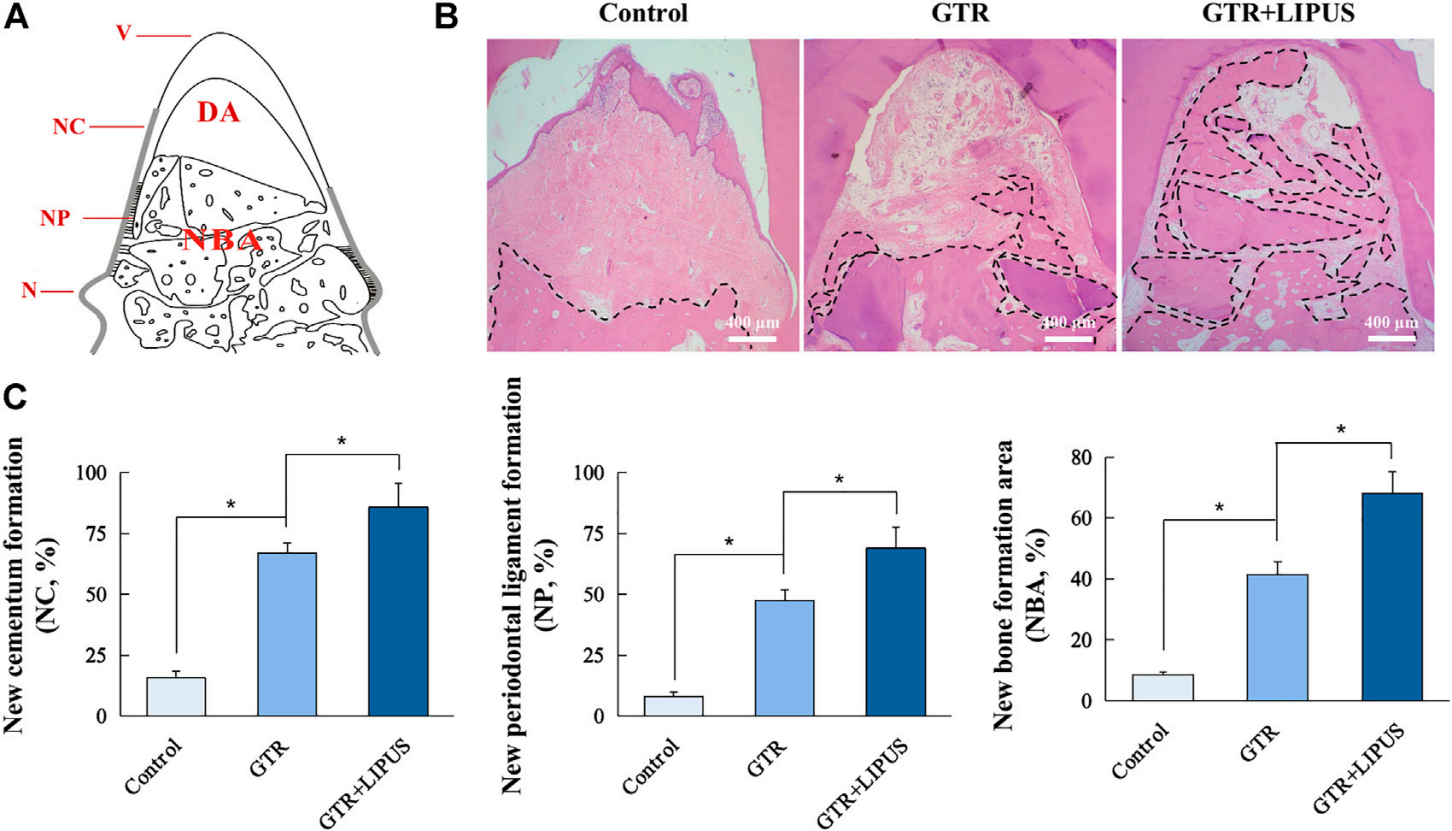
Histological and histomorphometric analyses. **(A)** Schematic illustration of histomorphometric measurements. N: notch; V: vertex of furcation; NC: new cementum; NC% = NC/NV×100%; NP: new periodontal ligament; NP% = NP/NV×100%; NBA: new bone area; DA: defect area; NBA% = NBA/DA×100%. **(B)** Representative H&E staining of the furcation area. The dotted lines indicate a new bone formation area. Scale bar, 400 μm. **(C)** Quantitative analysis of new cementum formation, periodontal ligament formation, and bone formation areas. Data are presented as mean ± SD, *p < 0.05.

**FIGURE 4:**
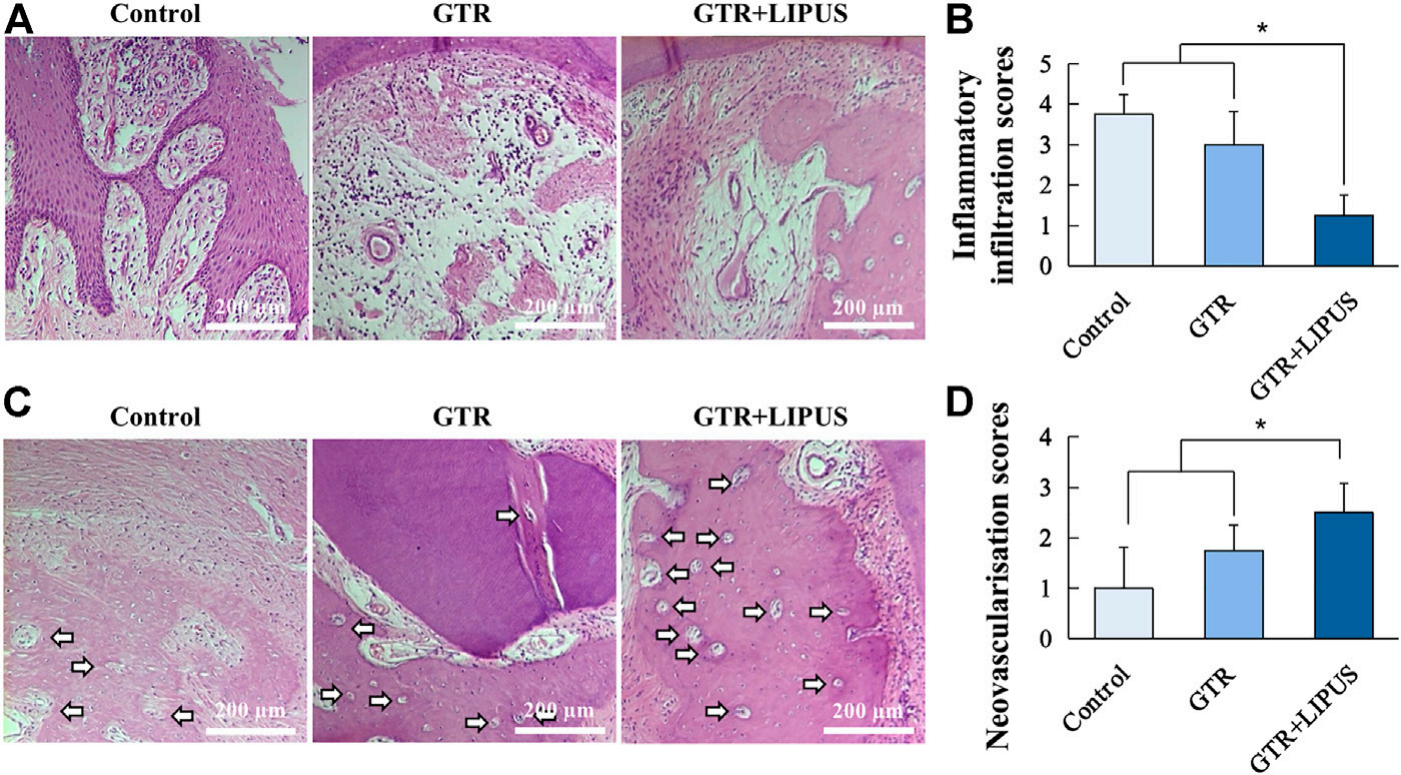
Histological assessment of inflammatory infiltration and neovascularization. **(A)** Lymphocyte infiltration of the control group, GTR group, and GTR+LIPUS group. Scale bar, 200 μm. **(B)** Inflammatory infiltration scores of different groups; *p < 0.05. **(C)** Blood vessels in the defect area are indicated by white arrows. Scale bar, 200 μm. **(D)** Neovascularization scores of different groups; *p < 0.05.

As shown in the H&E-stained sections (Figure 3B), there was limited new bone formation at the level of the apical notch in the control group, and most of the defect region was occupied by connective tissues. The section at higher magnification revealed evident epithelial hyperplasia and extensive lymphocyte infiltration in the control group, indicating severe inflammation status (Figure 4A). The semi-quantitative data of the control group from our previous work were re-analyzed (Figure 3C) (Ping et al., 2011). Compared to the control group, the newly regenerated alveolar bone in the GTR group was enhanced from 8.6% to 41.5%, respectively (Figure 3C). Additionally, coronal growth of newly formed periodontal ligament tissue and cementum-like structure on the dentine surface of the GTR group was also significantly improved compared to that of the control group (*p < 0.05). After LIPUS treatment, the newly regenerated periodontal tissues including alveolar bone, periodontal ligament tissue, and cementum-like structure were further increased (NBA: 68.1%, NP: 68.9%, and NC: 85.7%; *p < 0.05). Moreover, the semi-quantitative analysis of lymphocyte infiltration in the GTR + LIPUS group showed the lowest inflammation score (1.25 ± 0.50) among all the groups (*p < 0.05, Figure 4B). The neovascularization score of the GTR+LIPUS group was 42.9% higher than that of the GTR group (*p < 0.05, Figure 4D). It is suggested that the extent and distribution of lymphocyte infiltration in defects were alleviated by LIPUS (Figure 4A, Supplementary Figure S2), accompanied by the significant growth of micro-blood vessels in the newly formed bone matrix (Figure 4C, Supplementary Figure S3).

**FIGURE 5:**
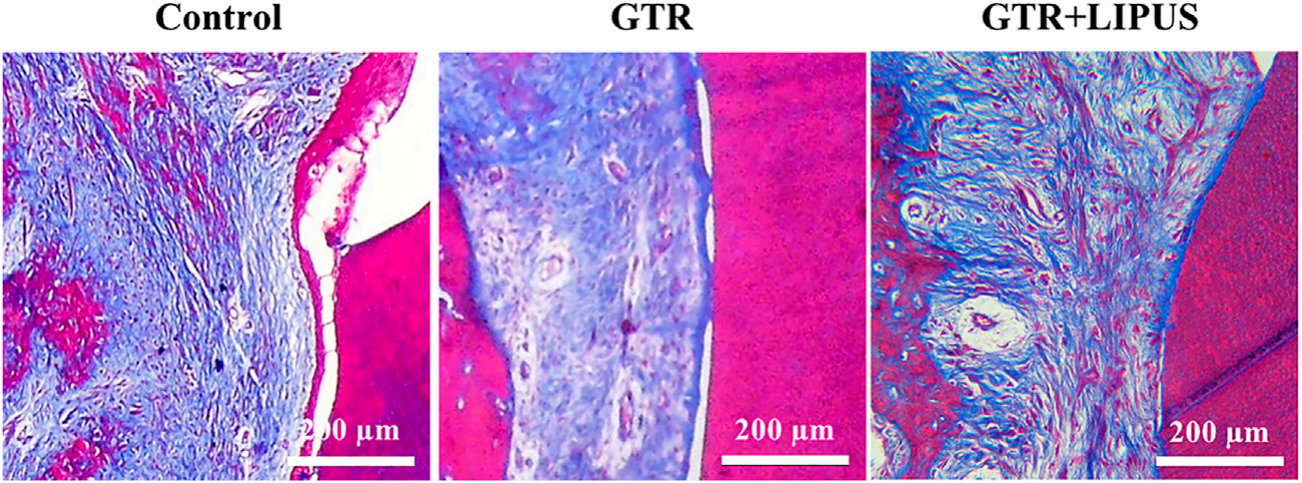
Histological assessment of periodontal attachment. Masson’s trichrome staining of the control group, GTR group, and GTR+LIPUS group. Scale bar, 200 μm.

The Masson’s trichrome staining (Figure 5, Supplementary Figure S4) displayed that the defect region of the control group was mostly filled with blue-dyed fibrous connective tissue. There was a thin layer of epithelium extending between the root surface and the connective tissue, indicating a healing pattern by the epithelial attachment. In the GTR+LIPUS group, more red-dyed bone matrix could be observed, indicating a relatively high maturity. Connective tissue repair (new attachment) was more easily spotted after LIPUS treatment, supported by the collagen fibers perpendicularly inserted into the cementum-like structure on the dentin surface.

## 4 Discussion

As a non-invasive modality, LIPUS has been widely applied in clinical treatment, such as promoting fracture healing, treating delayed union or nonunion fractures, and accelerating cartilage repair (Busse et al., 2009; Jiang et al., 2019). In recent years, growing evidence reveals that LIPUS therapy is also a promising strategy for the regeneration of periodontal tissue under inflammatory conditions (Kusuyama et al., 2019; Liu et al., 2019; Li et al., 2021). Although encouraging results were achieved, most studies focused on *in vitro* experiments, lacking direct evidence to support the therapeutic effect of LIPUS on the regeneration of periodontitis tissue. A beagle model of FI was utilized in our study to explore the *in vivo* effect of LIPUS on periodontal regeneration after GTR treatment. Our results showed that LIPUS not only exerted a positive impact on inflammation reduction and angiogenesis at the furcation region but also promoted new periodontal bone formation in the FI model after GTR treatment, providing preclinical evidence for the translational application of LIPUS.

It is well known that compared to healthy teeth, the treatment prognosis of teeth with periodontitis after GBR/GTR surgery is often unpredictable due to the prolonged healing period, which makes the diseased teeth exposed to various high-risk factors including secondary infection and poor oral hygiene (Jiang et al., 2018). One of the possible reasons for this phenomenon is that the function of PDLSCs, the cells responsible for periodontal regeneration, is compromised by chronic inflammation stimulation (Xu et al., 2019; Chen et al., 2021). Therefore, for an ideal periodontal regeneration after regenerative therapies, it is necessary to rescue the impaired function of PDLSCs by alleviating inflammation response in local periodontal tissue. Our previous work found that LIPUS could inhibit the expression of inflammatory factors in LPS-induced macrophages and PDLSCs (Zhang et al., 2017; Li et al., 2020). Later, Hsieh et al. (2018) revealed the decrease of lymphocyte infiltration in a post-traumatic osteoarthritis rat model owing to LIPUS treatment. More recently, Yu et al. (2021) unraveled that mechanical force may drive mesenchymal stem cells to endocytose inflammatory cytokines like TNF-α to maintain cellular homeostasis. Based on these previous studies, a hypothesis was proposed that the mechanical stimulation transmitted by LIPUS might have a potential impact on inflammation control on periodontitis tissue, which was further confirmed in our study. According to the results of histological analyses, the extent and distribution of lymphocyte infiltration in periodontal lesions, a typical feature of inflammation response, could be significantly attenuated by LIPUS treatment, suggesting the anti-inflammatory effect of LIPUS in periodontitis tissue. However, it is noted that changes in BI, PD, and AL were not statistically significant among all the groups. The possible explanation may be that the results of clinical assessment including BI, PD, and AL revealed the general periodontal status for buccal mesial, central, and distal sites of experimental teeth, while the newly regenerated periodontal tissue was mainly located at the root furcation region in treatment groups, so the therapeutic effect of GTR and LIPUS treatment on PD, AL, and BI at local sites (buccal central sites of experimental teeth) may not be revealed by the presented clinical assessment method in our study. Even so, the improved changes in PD, AL, and BI detected in three groups indicated the efficacy of routine root scaling prior to GTR and LIPUS treatment. Therefore, a more accurate histological evaluation could be achieved under identical periodontal hygiene conditions.

Vascularization in bone graft is regarded as one of the main prerequisites for successful periodontal regeneration (Saran et al., 2014; Rouwkema and Khademhosseini, 2016). Functional vascularization not only guarantees the nutrient supply and waste removal but also mediates the recruitment of osteoprogenitor cells and the delivery of osteo-regulatory factors (Maes and Clemens, 2014; Saran et al., 2014). Here, we found that the new bone matrix in the GBR+LIPUS group was superiorly vascularized, indicated by the highest neovascularization scores among the three groups. More importantly, the distribution of the newly formed vascular network was within 200 μm, which allows effective nutrient supply and waste removal, suggesting a pro-angiogenic effect of LIPUS. Shiraishi et al. (2011) previously revealed that LIPUS may improve angiogenesis in gingival tissue via the upregulation of connective tissue growth factors. Recently, it is reported that LIPUS could promote angiogenesis of human umbilical vein endothelial cells and vascular remodeling *via* YAP/TAZ activation (Xu et al., 2018). The evidence proved the angiogenic impact of LIPUS on soft tissue *in vitro*, but this effect on bone regeneration *in vivo* is rarely reported. Mechanistically, it is generally acknowledged that vascularization is largely influenced by the mechanical environment (Rouwkema and Khademhosseini, 2016; Kretschmer et al., 2021). Mechanical stimuli exert both a direct impact on endothelial cells and an indirect impact on the cell response to the vascular endothelial growth factor (VEGF) gradient (Mercado-Pagán et al., 2015). Lu et al. (2008) observed an early VEGF upregulation after LIPUS treatment at the patella–patellar tendon junction. Li et al. (2022) reported that LIPUS could enhance the mRNA level and secretion of VEGF in endothelial cells. As a pro-angiogenic cytokine, VEGF-mediated signaling has been demonstrated to have crosstalk with BMP signal transduction, which plays a critical role in the biofunctions of stem cells *in vivo* (Grosso et al., 2017; Putranti et al., 2022). Zhang et al. (2014) previously proved that the combined treatment with VEGF and BMP-2 could promote bone regeneration by facilitating endogenous stem cell homing and directing the differentiation of these cells into endothelial and osteogenic lineages. Therefore, we speculate that as a mechanical stimulus, LIPUS could accelerate perfusion in the bone graft and promote the “angiogenic–osteogenic coupling” process in the bone substitutes via regulating VEGF expression, which needs an in-depth investigation in future studies.

To date, various experimental animals have been explored to construct periodontal defect models, such as rodent, minipig, and dog. Among these animal models, rodent is considered to be a cost-efficient model for large-scale studies, but due to the narrow healing time window, different anatomical structures compared to humans, and technical challenges for creating furcation defect in small rodent models (Hector and William, 2010), large animals with similar histopathological features to human beings show more advantages as preclinical animal models for studying the safety and efficacy of medical devices targeting periodontal regeneration. Therefore, the beagle model of FI was employed in our study to explore the *in vivo* effects of LIPUS on periodontitis tissue (Ribitsch et al., 2020). Our results revealed that LIPUS could accelerate the maturation of the bone matrix. Moreover, compared to control groups, the amount of newly formed cementum, periodontal ligament, and alveolar bone tissues at the furcation defect region was significantly elevated in the LIPUS-treated group at a six-week healing interval, suggesting a positive effect of LIPUS on the regeneration of periodontitis tissue, which may be ascribed to the previous findings that LIPUS could rescue the impaired function of PDLSCs like proliferation, migration, and osteogenic capacity under inflammatory conditions. Additionally, the anti-inflammatory and pro-angiogenic effects of LIPUS may also contribute to the establishment of a suitable microenvironment for enhanced biofunction of stem cells. Although encouraging results were achieved *in vivo*, the translational application of LIPUS from laboratory to clinical practice still faces many challenges because the current translation parameters such as ultrasonic intensity, frequency, wave width, and pulse ratio for LIPUS treatment in periodontal regeneration were mainly based on the results from *in vitro* research studies, but during the *in vivo* transmission of ultrasonic waves, the therapeutic signals may be disturbed by various factors including the thickness of soft tissue, barrier membranes, bone graft materials, and special anatomical structures in the periodontal region. Therefore, to obtain the optimal treatment outcome, more efforts are required in the future to parameterize LIPUS for clinical translational application.

In conclusion, the present study provides preclinical evidence to prove the therapeutic effect of LIPUS as an adjunctive therapy for periodontal regeneration in a beagle model of FI, suggesting a promising potential for LIPUS application in the dental field.

## Data Availability

The original contributions presented in the study are included in the article/Supplementary Material; further inquiries can be directed to the corresponding authors.
