# Supplementary figures and images for: Low-intensity pulsed ultrasound promotes periodontal regeneration in a beagle model of furcation involvement
Source: Front Bioeng Biotechnol. 2022 Aug 26;10:961898. doi: 10.3389/fbioe.2022.961898 (PMC9458930; doi:10.3389/fbioe.2022.961898)

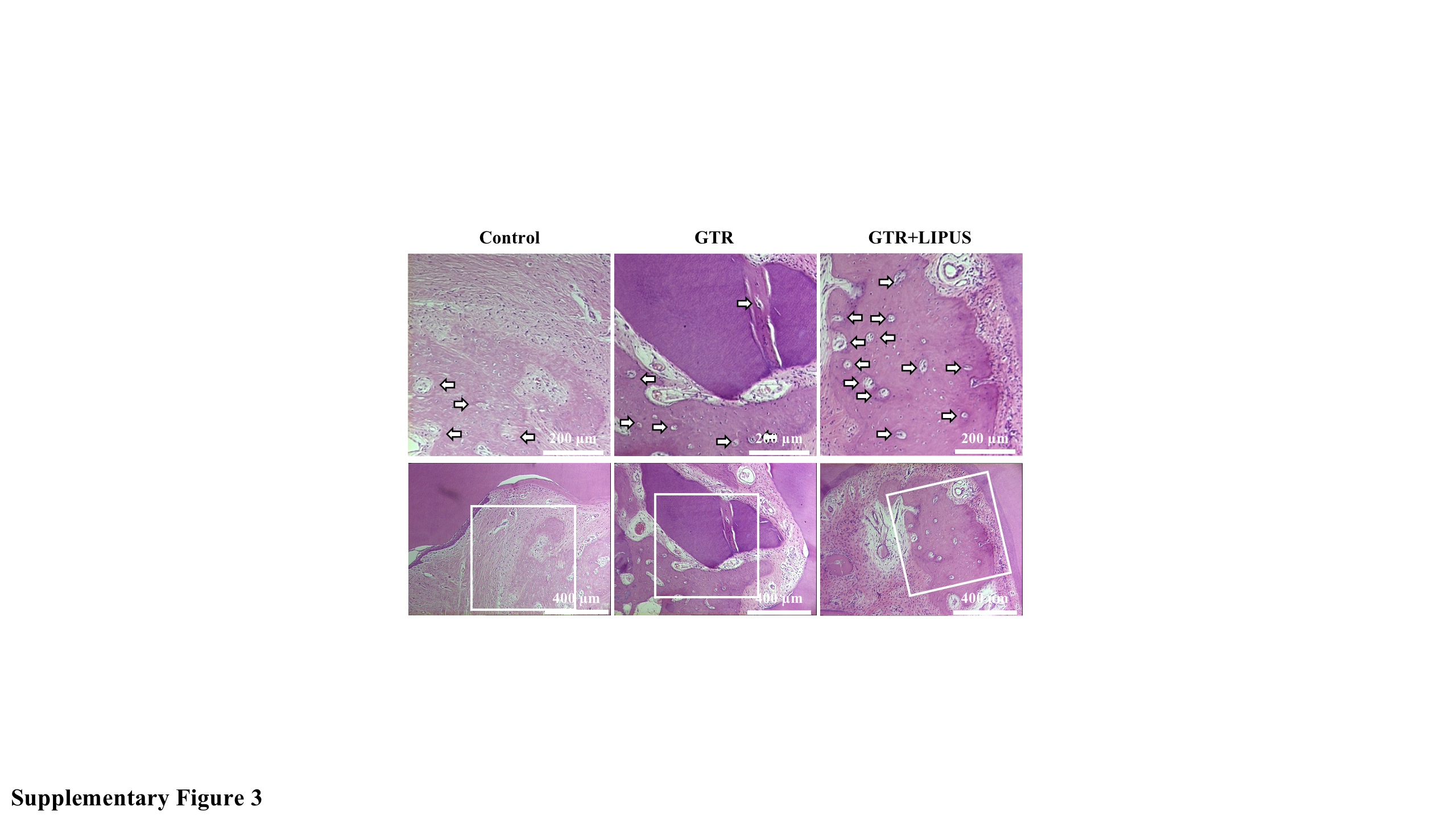

Supplement: Supplementary file 1 [file Image3.TIF]

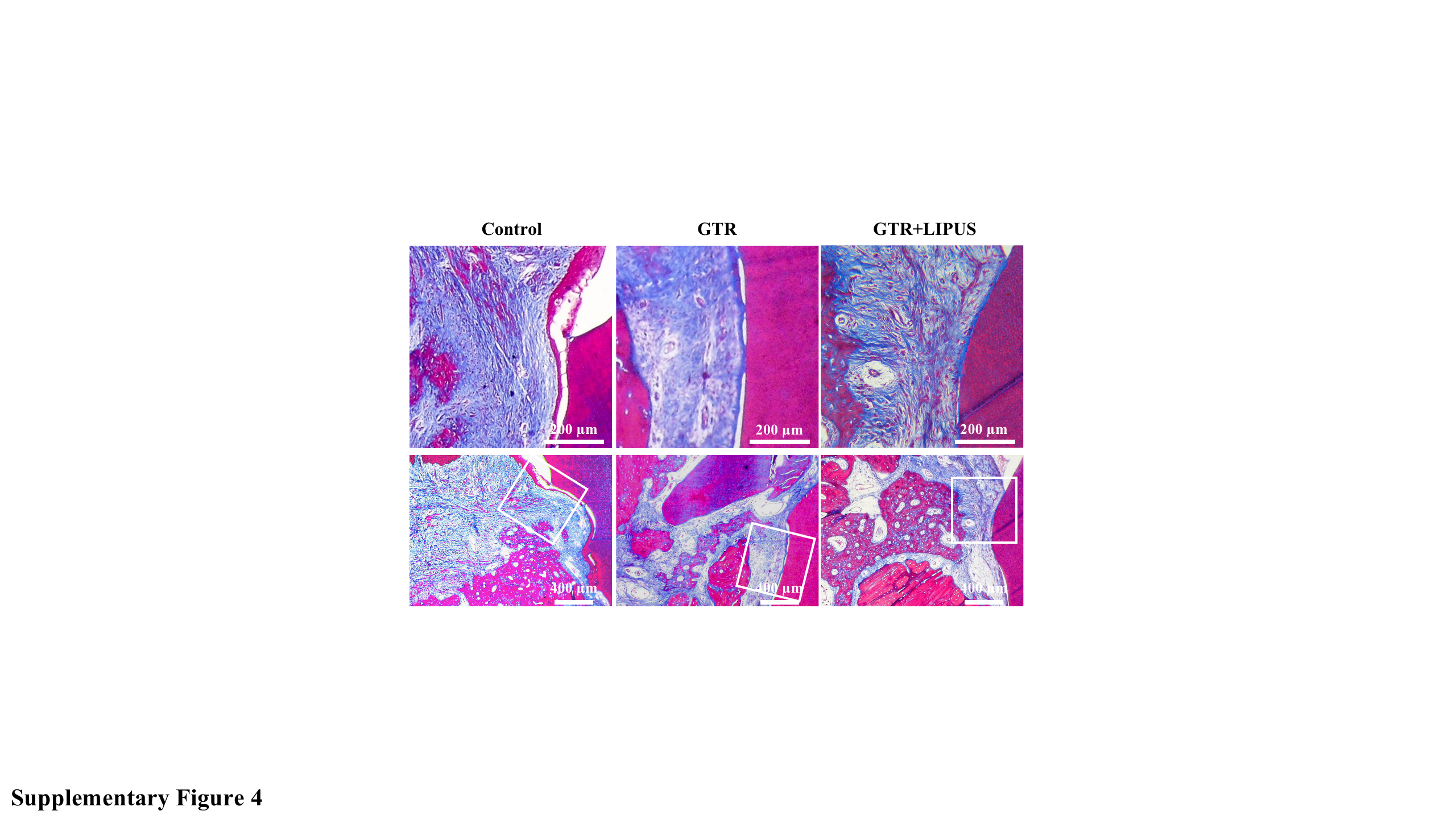

Supplement: Supplementary file 2 [file Image4.TIF]

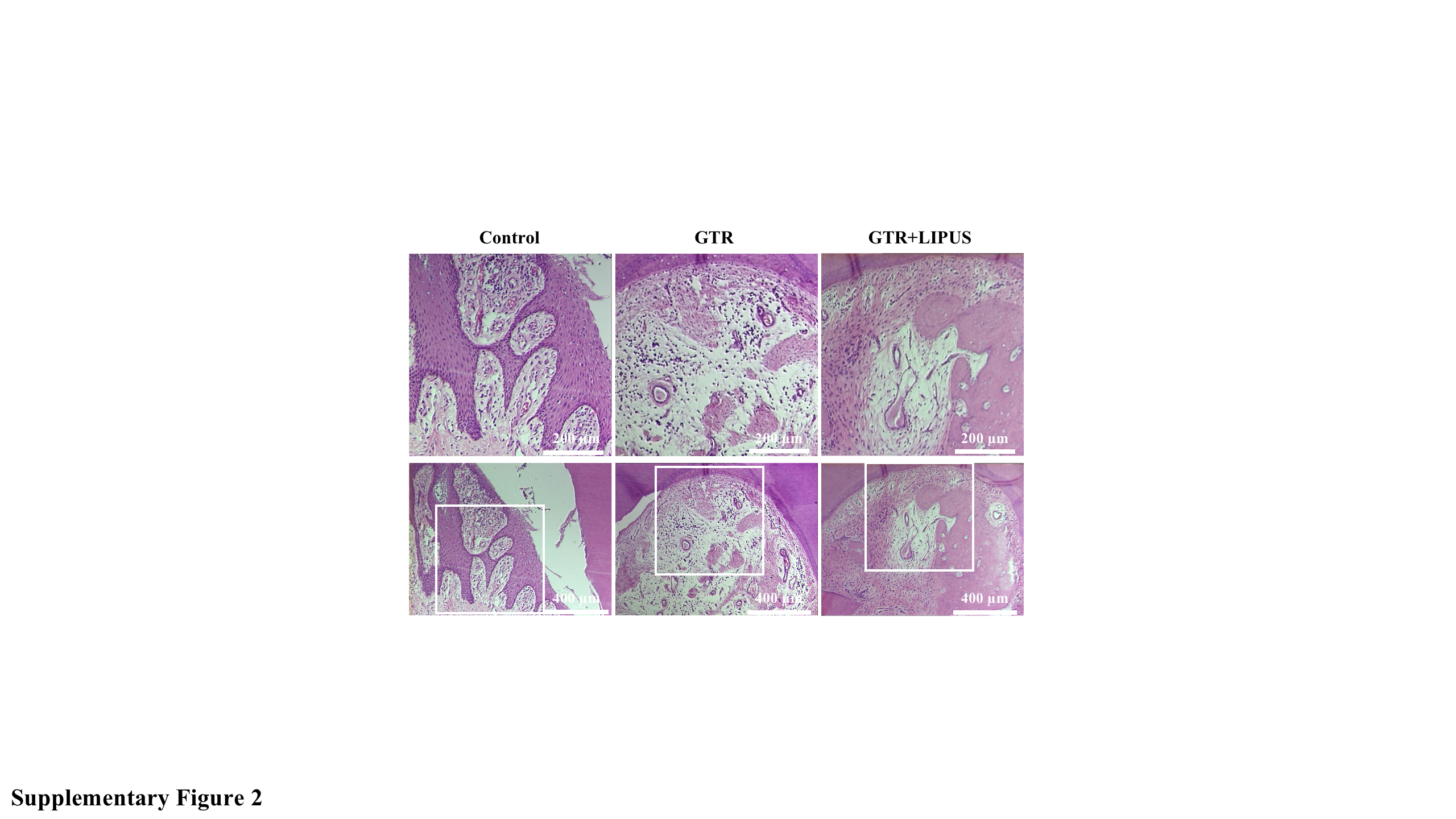

Supplement: Supplementary file 3 [file Image2.TIF]

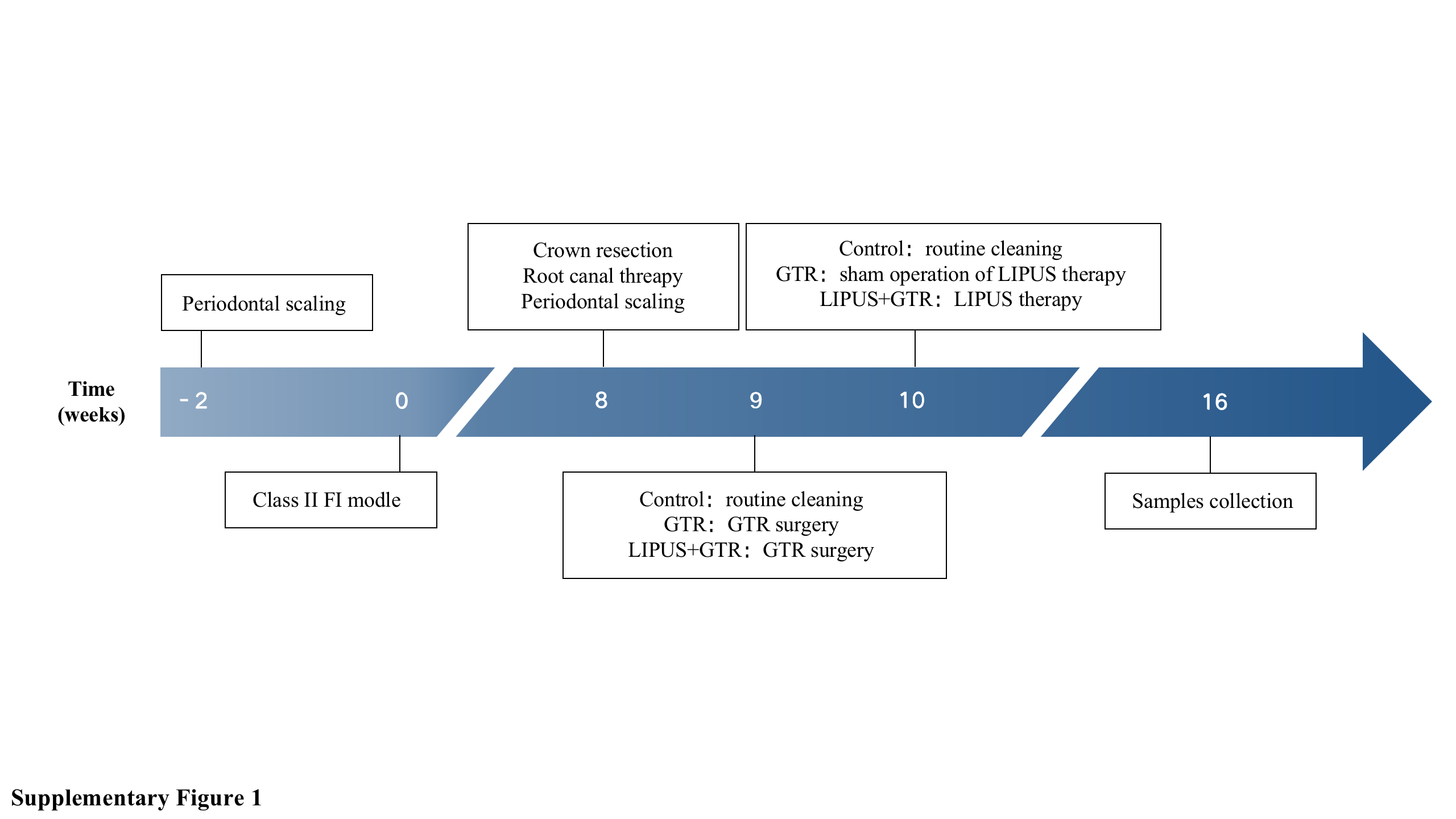

Supplement: Supplementary file 4 [file Image1.TIF]
