# Supplementary material for: Low-intensity pulsed ultrasound promotes periodontal regeneration in a beagle model of furcation involvement
Source: Front Bioeng Biotechnol. 2022 Aug 26;10:961898. doi: 10.3389/fbioe.2022.961898 (PMC9458930; doi:10.3389/fbioe.2022.961898)
Supplement: Supplementary file 5 [file DataSheet1.PDF]

Supporting Information

Low intensity pulsed ultrasound promotes periodontal regeneration in a beagle model of furcation involvement

Yue Wang [1, 2, 3†], Qingyue Xiao [1, 2, 3†], Wenjie Zhong [1, 2, 3], Chuangwei Zhang [1, 2, 3], Yuanyuan Yin [1, 2, 3], Xiang Gao [1, 2, 3*], and Jinlin Song [1, 2, 3*]

[1]College of Stomatology, Chongqing Medical University, Chongqing 401147, China

[2]Chongqing Key Laboratory of Oral Diseases and Biomedical Sciences, Chongqing 401147, China

[3]Chongqing Municipal Key Laboratory of Oral Biomedical Engineering of Higher Education, Chongqing 401147, China

Corresponding Author

*Prof. Xiang Gao, Chongqing Key Laboratory for Oral Diseases and Biomedical Sciences, Chongqing 401147, China

Tel & Fax: +86 23 88860105. E-mail: xiangg@hospital.cqmu.edu.cn

*Prof. Jinlin Song, Chongqing Key Laboratory for Oral Diseases and Biomedical Sciences, Chongqing 401147, China

Tel & Fax: +86 23 88860026. E-mail: songjinlin@hospital.cqmu.edu.cn

Author Contributions

[#]Authors Yue Wang and Qingyue Xiao contributed equally to this work. The manuscript was written through contributions of all authors. All authors have given approval to the final version of the manuscript.

Notes

The authors declare no competing financial interest.

Keywords: furcation involvement, low-intensity pulsed ultrasound, inflammation, regeneration, beagle dog

Figure S1. The experimental design and timeline.

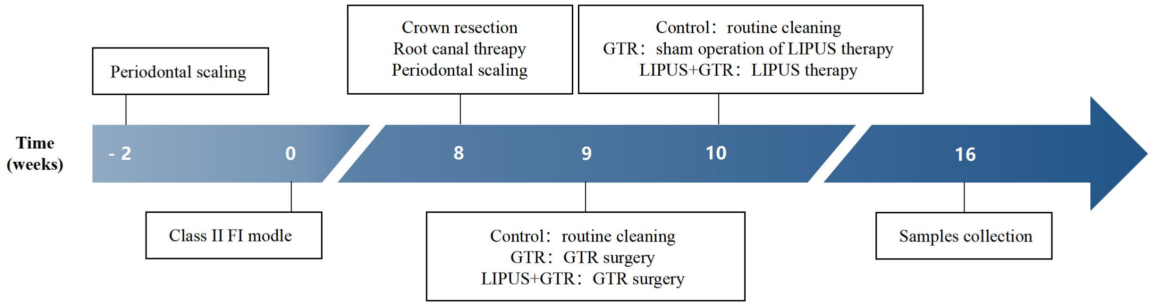

Figure S2. The low magnification images of lymphocytes infiltration.

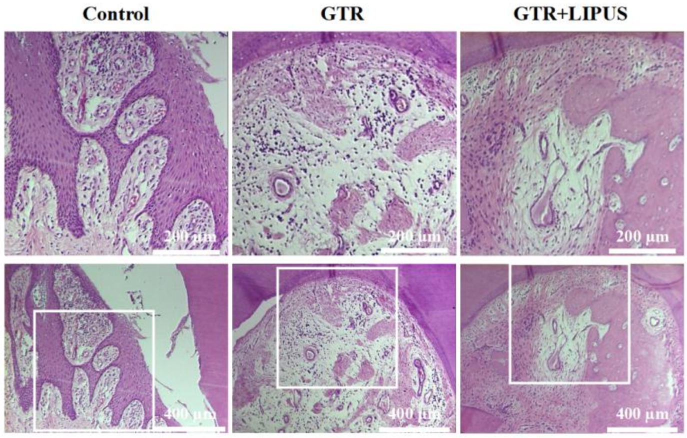

Figure S3. The low magnification images of blood vessels.

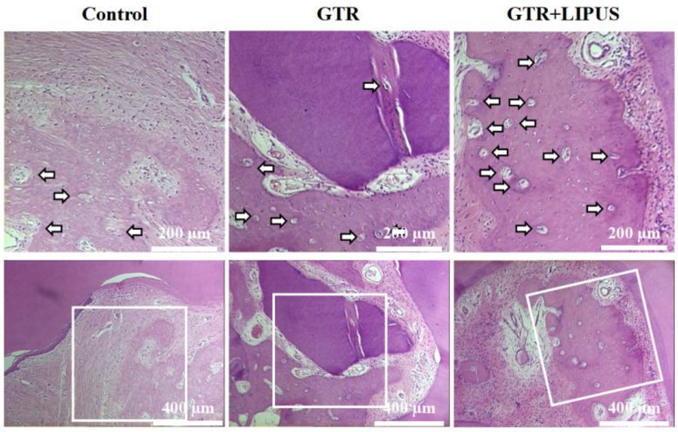

Figure S4. The low magnification images of periodontal attachment.

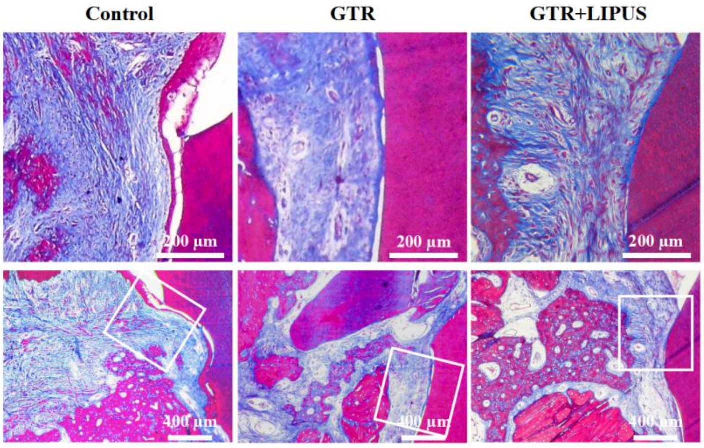

Table S1. Semi-quantitative scoring system for inflammatory infiltration.

Cell type	Score				
	0	1	2	3	4
lymphocytes	0	Rare	Mild	Heavy infiltrate	Packed

Table S2. Semi-quantitative scoring system for neovascularization.

Response	Score				
	0	1	2	3	4
Neovascularization	0	1-3 mild capillaries proliferation	4-7 capillaries proliferation	Broad band of capillaries	Extensive band of capillaries
